# Using Sensorized Gloves and Dimensional Reduction for Hand Function Assessment of Patients with Osteoarthritis

**DOI:** 10.3390/s21237897

**Published:** 2021-11-26

**Authors:** Verónica Gracia-Ibáñez, Pablo-Jesús Rodríguez-Cervantes, Vicente Bayarri-Porcar, Pablo Granell, Margarita Vergara, Joaquín-Luis Sancho-Bru

**Affiliations:** 1 Department of Mechanical Engineering and Construction, Universitat Jaume I, 12071 Castelló de la Plana, Spain; cervante@uji.es (P.-J.R.-C.); vbayarri@uji.es (V.B.-P.); vergara@uji.es (M.V.); sancho@uji.es (J.-L.S.-B.); 2 Consorci Hospitalari Provincial de Castelló, Av. del Dr. Clarà, 19, 12002 Castelló de la Plana, Spain; pablo.granell@hospitalprovincial.es

**Keywords:** hand osteoarthritis, hand function assessment, kinematics reduction, kinematic coordination, principal component analysis

## Abstract

Sensorized gloves allow the measurement of all hand kinematics that are essential for daily functionality. However, they are scarcely used by clinicians, mainly because of the difficulty of analyzing all joint angles simultaneously. This study aims to render this analysis easier in order to enable the applicability of the early detection of hand osteoarthritis (HOA) and the identification of indicators of dysfunction. Dimensional reduction was used to compare kinematics (16 angles) of HOA patients and healthy subjects while performing the tasks of the Sollerman hand function test (SHFT). Five synergies were identified by using principal component (PC) analyses, patients using less fingers arch, higher palm arching, and a more independent thumb abduction. The healthy PCs, explaining 70% of patients’ data variance, were used to transform the set of angles of both samples into five reduced variables (RVs): fingers arch, hand closure, thumb-index pinch, forced thumb opposition, and palmar arching. Significant differences between samples were identified in the ranges of movement of most of the RVs and in the median values of hand closure and thumb opposition. A discriminant function for the detection of HOA, based in RVs, is provided, with a success rate of detection higher than that of the SHFT. The temporal profiles of the RVs in two tasks were also compared, showing their potentiality as dysfunction indicators. Finally, reducing the number of sensors to only one sensor per synergy was explored through a linear regression, resulting in a mean error of 7.0°.

## 1. Introduction

The human hand has complex kinematics provided by 19 joints, some of them with various degrees of freedom (DoF). This complexity is essential in enabling daily function and human autonomy. The measurement of hand kinematics can provide useful information for the objective assessment of hand function [1]. In this regard, the World Health Organization established that such assessment should be based on the objective evaluation of the hand’s capability to perform activities of daily living (ADL) [2]. However, ADL performance is rarely assessed in current clinical practice but is instead assessed by means of subjective questionnaires [3,4,5]. Some standardized tests consisting in performing simulated ADL have been proposed in the past for an objective assessment of ADL performance [6,7,8]. The Sollerman hand function test (SHFT) [7] has the most varied and representative set of ADL. However, the test score considers only the time of accomplishment of each task and the grasp type used and has a highly subjective component depending on the operator who performs the visual assessment [9]. Monitoring kinematics during a performance of this test has been proposed as a source of accurate and objective information with respect to motor strategies associated with goal-oriented tasks, in addition to allowing for better supervision of the administration of therapeutic techniques [9].

Hand osteoarthritis (HOA) is a chronic disorder causing pain and mobility limitation that may affect hand function, with a high prevalence especially in women over 50 years of age. Reduction in the active range of motion (AROM) has been reported [10,11,12], causing hand disability [13,14]. Notwithstanding, HOA is clinically treated only in very severe situations, and it is usually unnoticed by clinicians in most cases, even though applying adequate treatments in early stages would benefit patients’ quality of life while preventing structural progression of the disease [15]. Studies measuring hand kinematics of HOA patients during ADL performance are scarce and limited to the recording of only a few activities and joints [11,16,17]. Holland et al. [11] used videogrammetry to study the thumb and index finger joints during five ADL by recording a representative static posture from each task. Luker et al. [16] also used videogrammetry but only recorded the kinematics of the thumb of one HOA patient during three ADL. Tanashi et al. [17] used an electromagnetic tracking system to compare the kinematics of the thumb, index, and middle fingers of nine patients while performing nine ADLs with and without joint protection strategies and assistive devices. This scarcity of studies is probably associated with the difficulty of simultaneously recording the high number of DoF of the hand during manipulative activities: videogrammetry and other optical systems such as Kinect have occultation problems during object manipulation [18], electromagnetic devices are altered by metallic components [19], and inertial systems are still too large for monitoring all hand segments.

There are commercially available instrumented gloves, such as Cyberglove (Cyberglove Systems LLC; San Jose, CA, USA), that can overcome these problems, but they have traditionally required long and tedious calibration protocols for each subject in order to achieve good accuracy [20,21,22,23]. In a recent study, the authors proposed the use of an across-subject calibration so that once calibrated for a set of subjects, only the measurement of a reference posture for each subject is required to be able to record data [24]. Furthermore, the glove has proven to be suitable for recording tasks requiring medium and gross manipulation skills [25] and has been successfully used for the characterization of the kinematics of healthy subjects [25,26]. However, it has not yet been used to characterize the kinematics of HOA patients.

One important obstacle hindering monitoring hand kinematics in clinical practice lies in the difficulty of analyzing the high number of DoF that are used simultaneously while performing ADL. Biomechanical indicators need to be identified to make interpretation of the data easier. Another main obstacle lies in the affordability of the mocap systems. The concept of synergies can help to overcome both obstacles. Kinematic synergies [27] are suggested as a method for representing the basic building blocks underlying natural hand motions that can be used to reduce the dimensionality of hand kinematics. Principal component analysis (PCA) is the most widely used method for dimensionality reduction. PCA looks for linear combinations of correlated variables to find a small set of new uncorrelated variables, the principal components (PCs), that describe most data variation [27,28]. Each PC is a vector containing the loadings by which each original variable should be multiplied by in order to obtain the new variable and represents a kinematic synergy.

PCA has been extensively applied to the analysis of static postures [29], reach-to-grasp movements [30,31], and manipulative tasks [32]. Recent studies have also tested its effectiveness in reconstructing the entire healthy hand kinematics [33,34]. However, studies analyzing hand kinematic synergies during representative tasks of daily activities are limited to previous studies of the authors [26,35], who used PCA on kinematics recordings of healthy subjects during the performance of standardized [26] and non-standardized tasks [35,36], reducing the problem to the study of five coordinated synergies. Moreover, the authors’ previous exploratory studies on several patients seem to indicate the existence of alterations in kinematic synergies [37,38,39]. The identification of these alterations might help in clinical diagnoses or function assessments of the HOA hand, but it requires the study of representative samples of HOA patients and healthy subjects during the performance of representative ADL. The search of biomechanical indicators using kinematic synergies has the advantage of using entire hand kinematics to detect alterations, and not only those of some joints but as the kinematics are reduced to a small set of variables, the comparison can be performed in an easier and more global method by looking at biomechanical indicators. Furthermore, the observed kinematic synergies could also be applied to reconstruct entire hand kinematics from the recording of only a few joint angles by estimating the remaining angles from the coordination established by those synergies. This has already been proven to be feasible in healthy subjects [40] but needs to be studied in patients. Lowering the number of hand joints to be recorded would reduce the investment required.

Therefore, the aim of this study is to propose more feasible alternatives for using the kinematics of the entire hand to diagnose and assess the hand function of HOA patients in clinical practice. We used an instrumented glove to measure the kinematics of the right (dominant) hands of twenty-seven healthy subjects and thirty-three HOA patients, while performing the activities of the SHFT. The underlying kinematic synergies were obtained in both groups and were used to study the strategies followed by patients to make up for their deficiencies. The kinematics of both groups were compared in terms of reduced variables (RVs) in order to look for indicators of kinematic alterations that were used later to identify a discriminant function for the detection of HOA. Finally, the results were used to explore a reduction in the number of sensors required to record the hand kinematics of HOA patients.

## 2. Materials and Methods

### 2.1. Experimental Study

**Figure 1:**
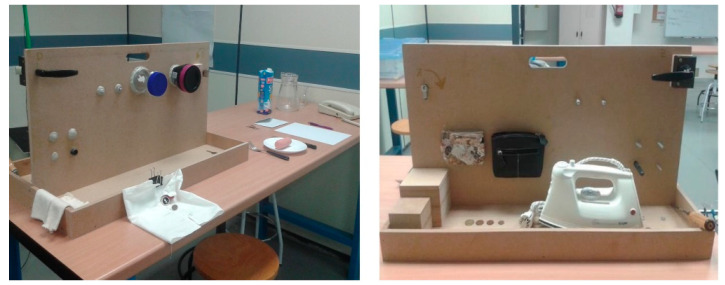
Scenarios with the objects used in SHFT.

Twenty-seven right-handed healthy adults (14 females and 13 males, 38.70 ± 7.60 years) and thirty-three right-handed adults with HOA (all females, 70.12 ± 9.53 years) participated in the experiment. HOA patients showing different stages of the disease and different levels of compromise were recruited by clinicians: 49% presented mild to moderate disorders, 24% moderate to severe and 27% severe, and none of them underwent surgery. The healthy subjects (control group) were free from upper limb pathologies or injuries. The hand kinematics of both samples were recorded while performing the tasks of SHFT, which is based on the most common hand grips and consists of 20 ADL [7] (Table 1, Figure 1). Each subject performed these 20 ADL under laboratory conditions following the test instructions strictly and by using real objects. All the participants provided their informed consent to participate in the experiment (approved by the Hospital and by the University Ethics Committees, reference numbers CD/31/2019 and CD/65/2020), and specific informed consent for publication in an online open-access publication was obtained for photos that could allow the identification of the participant. Finally, patients were asked to report any difficulties they had when carrying out ADLs at home.

**Figure 2:**
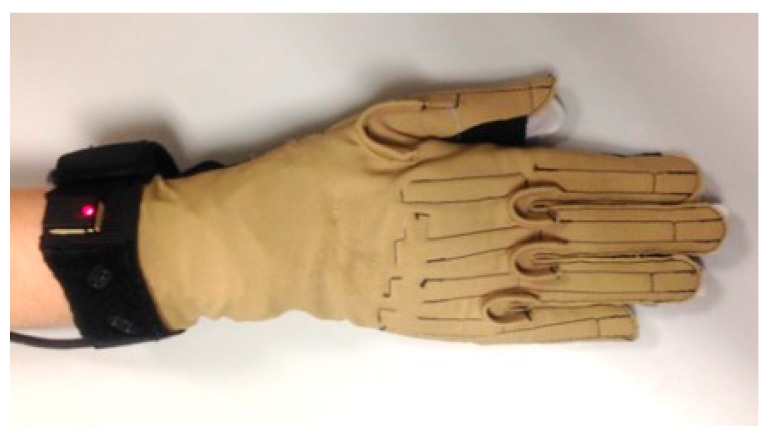
Reference posture considered with the instrumented glove (0°).

Sixteen joint angles were recorded (100 Hz) with an instrumented glove (Cyberglove Systems LLC; San Jose, CA (USA)) (Figure 2) using a validated calibration protocol [24]: flexion of metacarpophalangeal joints (MCP1 to MCP5, 1 to 5 meaning thumb to little digits), flexion of interphalangeal thumb joint (IP1), flexion of proximal interphalangeal joints of the fingers (PIP2 to PIP5), flexion and abduction of the carpometacarpal thumb joint (CMC1), relative abduction between finger MCPs (index-middle, middle-ring, and ring-little), and palmar arching. Flexion and abduction angles were considered to be positive. The recordings were filtered with a 2nd-order 2-way low-pass Butterworth filter with a cut-off frequency of 5 Hz. Each SHFT task was resampled to 1000 frames so that all tasks weighed the same when looking for underlying synergies. Therefore, the data used throughout all the paper consist of 20 records of 1000 frames for each participant.

### 2.2. Data Analysis

#### 2.2.1. Kinematic Synergies of Hand Joints in Healthy and HOA Samples

For each sample, after checking the appropriateness of PCA through a Bartlett’s test of sphericity, a PCA was applied to the 16 joint angles measured in all the records, each record containing the entire kinematic time series recorded. First, joint angles were standardized (mean = 0; standard deviation = 1) to make angles with different ranges of motion comparable [41,42]. The correlation matrix was then computed and used to calculate eigenvalues (variance explained) and eigenvectors (principal components). To simplify the interpretation of the PCs, Varimax rotation [42] was performed, i.e., the sum of the variances of the squared loadings was maximized so that each PC comprises only a few variables with very high loadings on this PC, while the remaining variables have near-zero loadings. The rotated PCs with eigenvalues greater than 1 in each sample were used as kinematic synergies. The variance explained by the PCs in each PCA was used as an indicator of how well the measured motion fitted the coordination represented by the PCs obtained.

Therefore, two sets of PCs were obtained: one for healthy subjects (HPCs) and one for pathologic subjects (PPCs). Similarity of synergies between HPCs and PPCs was evaluated through the angle (absolute cosine) between synergies.

In order to propose a set of indicators to be used for quantifying the effect of hand kinematics on hand function in HOA patients, the resulting HPCs were considered as a normal reference, and different analyses were performed, as detailed in the next two sections.

#### 2.2.2. Can HPCs Be Used for Patients?

The PCs found for the healthy sample (HPCs) were obtained in a global analysis with the data of all the subjects together. In order to check whether these HPCs can be used for dimensional reductions in a particular subject (healthy or patient), the variance explained by these HPCs was calculated for the data of each subject from both samples. The purpose is to check whether these HPCs can substitute the original joint angles, in order to reduce the dimensionality of the problem.

#### 2.2.3. How Reduced Kinematics Can Help Assess HOA Pathology?

Once HPCs are checked for their utilization, indicators of kinematic alterations in HOA patients were investigated by comparing the kinematics of HOA patients versus healthy subjects in terms of the HPCs. The scores corresponding to the HPCs were considered as the new set of reduced variables (RVs). For each record of each subject (both healthy and patients), the values of new RVs were calculated at each of the 1000 resampled frames. The comparison was conducted in two ways: using summarizing parameters of the RVs for each participant and considering time evolution of RVs during the tasks.

First, as a method to summarize the frames for each participant, median, 5th percentile (p5), 95th percentile (p95), and range (p95–p5) of the 20 records altogether were computed for each reduced variable (RVi), and statistics across subjects of these summarizing parameters were obtained. Additionally, after applying Shapiro–Wilks to test normality, a set of ANOVAs was applied for each summarizing parameter of each RVi, with the sample as the factor, in order to check for significant differences between samples.

Then, a linear discriminant analysis was performed, aimed at locating a reduced set of predictive parameters for detecting HOA. The statistics of the RVi that presented significant differences in the previous ANOVAs were considered independent variables, and the condition (HOA patient vs. healthy subject) was considered as the grouping variable. The stepwise method was used (predictors entered sequentially), which searches for the highest correlated predictors. In particular, the Wilks’ lambda was used, which checks how well each independent variable (potential predictor) contributes to the model: 0 means total discrimination, and 1 means no discrimination. Each independent variable is tested by placing it into the model and then taking it out, generating a Λ statistic. The significance of the change in Λ is measured with an F-test. The variable is entered into the model if the significance level of its F value is less than the entry value (0.05), and it is removed if the significance level is greater than the removal value (0.1). The goodness of the classification ability was checked by means of a leave-one-out cross validation, which repeats the analysis by taking one case out in each repetition. In addition, percentage of the patients correctly and incorrectly classified was checked.

The prediction ability of the discriminant function obtained was compared to the prediction ability of the SHFT by running an analogous linear discriminant analysis, but using the SHFT scores and total times of performance as independent variables. To perform this, the time score was normalized to render it non-dependent on age and gender by using the data of time scores per gender and age from [43]. The normalized time score for each subject was obtained by multiplying the time scores by the maximum normative time for any age of those of the same gender as the subject and dividing by the normative value for subject’s gender and age. SHFT scores cannot be corrected for these differences, because there are no normative data available to perform this correction.

Second, for the comparison of time evolution of RVs of patients versus those of healthy subjects, the mean posture across subjects (with a 95% confidence interval) of each RVi of both samples was graphically represented versus frame in two representative tasks, searching for detectable differences that could be considered for further analysis as indicators of alterations in the kinematics. Two tasks were selected: one as representative of gross manipulation (pour water from a jar), and a second one that is representative of fine manipulation (cut Play-Doh with a knife and fork), this last one reported being by patients as problematic for their daily lives.

#### 2.2.4. Reducing the Number of Sensors

As a method to reduce the complexity (and thus price) of equipment needed to apply the results of this study in clinical practice, kinematics synergies obtained for patients (PPCs) were used to explore the feasibility of reducing the number of sensors to record entire hand kinematics. The angle with the highest loading in each synergy was used to compound the proposed set of joint angles to be measured in patients. Due to Varimax rotation, if a sensor appears with very high loading in a PC, it will have near-zero loadings in other PCs. Therefore, choosing the sensor with the highest loading from each synergy provides a set of sensors that produce quite independent information.

The exploration of the feasibility of using only this reduced set of joint angles has been carried out by (1) estimating the rest of the joint angles through a linear regression and (2) computing the errors from the estimation through the mean residual standard deviations (MRSDs) in a general univariate linear model with ‘Subject’ and ‘Task’ as factors.

## 3. Results

### 3.1. Data Analysis

#### 3.1.1. Kinematic Synergies of Hand Joints in Healthy and HOA Samples

The Bartlett test of sphericity confirmed the appropriateness of the PCA (significance level < 0.05 for each sample). Table 2 shows the results of the PCAs performed on the joint angles in the two samples. Five PCs were obtained in both cases, which explain 72.74% of the data variance in healthy subjects and 71.62% in HOA patients. The coordinations represented by these kinematic synergies can be observed from the loadings (higher values in a PC mean more coordinated joint movements, and a negative sign means opposite movement coordination). Table 3 shows the similarity of synergies between samples from the angles between the corresponding PCs (lower angles represent more similarity, and angles close to 90° represent no similarity at all). The first two synergies in both samples are similar (angles below 20 degrees), although they are interchanged: HPC1 and PPC2 are the coordinated flexions of the PIP joints of fingers (i.e., *fingers arch*), explaining less variance in patients; and HPC2 and PPC1, with similar variance explained in both samples, are the coordinated flexions and adduction of MCP joints of fingers (i.e., *hand closure*). The other synergies (higher order PCs) in both samples explain much less variance and present less similarity between samples. The third synergy in healthy subjects (HPC3) mostly depicts the coordination of thumb joints and index MCP flexion (i.e., *thumb-index pinch*) and has no clear correspondence with patients’ synergies (all angles above 50 degrees). The fourth synergy in healthy subjects (HPC4) shows a coordinated abduction of the thumb CMC joint with flexion of the IP joint and extension of the MCP joint, which appears in the case of force applied on the thumb tip (i.e., *forced thumb opposition*) and presents its lowest angle (41 degrees) with the fifth synergy of patients (PPC5), where the abduction of the thumb CMC joint is substituted by CMC extension. More independent thumb abduction is depicted by PPC4 in patients. The fifth synergy in healthy subjects (HPC5) is the coordination of palmar arch and thumb CMC abduction (i.e., *palmar arching*), which presents its lowest angle (48 degrees) with the third synergy of patients. Palmar arching acquires more relevance in patients, with higher variance explained so that it appears in the third position instead of the fifth position.

#### 3.1.2. Can HPCs Be Used for Patients?

**Figure 3:**
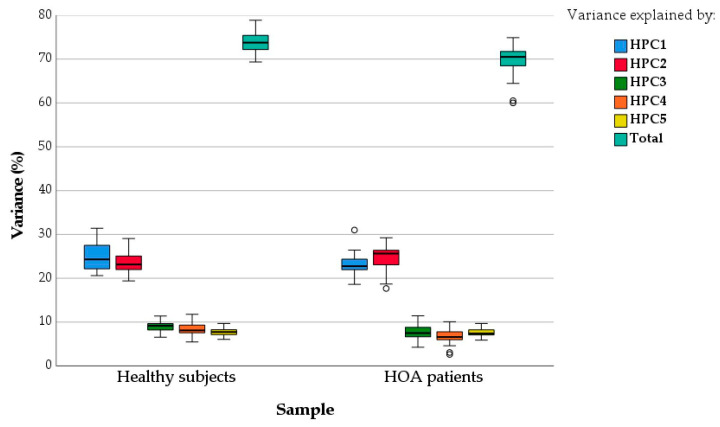
Variance explained (%) by each HPC and by all HPCs (Total), calculated for every subject in each sample (box and whisker plot).

Figure 3 shows the box and whisker plot for the variance explained by each HPC and by all HPCs (Total), which was calculated for every subject in both samples. The total amount of variance explained by HPCs in both samples is similar, although slightly it was smaller in patients (70 (±3) vs. 74 (±2)). The HPCs can, thus, not only be used for the dimensional reduction in kinematic data of the healthy subjects but also of HOA patients.

#### 3.1.3. How Reduced Kinematics Can Help Assess HOA Pathology?

Comparison of the kinematics of healthy and HOA samples by using reduced variables (RVi) can be observed in Table 4, which presents the statistics (mean, standard deviation, and minimum and maximum values) of each RVi summarizing value (Median, 5th percentile, 95th percentile, and range). Those RVi summarizing values for which significant differences (level of significance 0.05) were found between samples in the ANOVAs are marked with a tick and were used for the discriminant analysis afterwards.

From the twelve independent variables used in the step-by-step discriminant analysis, only three were finally introduced in the model (Equation (1)). The discriminant scores found were able to predict the assignment of subjects participating in the experiment with a success ratio of 85% (85.2% for healthy subjects and 84.8% for patients). The success ratio after cross validation was 81.7% (85.2% for healthy subjects and 78.8% for patients). The values of the predictive parameters can be used to calculate discriminant scores F for each subject, according to Equation (1), so that when F is positive, the prediction is that the subject is healthy, and if F is negative, the subject has HOA. All the ‘moderate to severe’ and ‘severe’ patients and most of the ‘mild to moderate’ ones were correctly classified by the analysis.

(1) F=1.591·RV1range+1.274·RV3range+1.278·RV4range−19.720

Discriminant analysis using SHFT scores and normalized total times of performance as independent variables provided a predictive success rate of only 57.6% when assigning patients. SHFT results obtained by healthy subjects/HOA patients were as follows: 76 ± 2/68 ± 10 in scores and 248 s ± 44 s/295 s ± 107 s in total normalized time. HOA patients could accomplish SHFT practically with no difficulties. However, most of them reported difficulties in their daily lives for handling very small objects, for handling heavy objects and in the specific tasks of opening jars (tightly closed), and in cutting with a knife.

**Figure 4:**
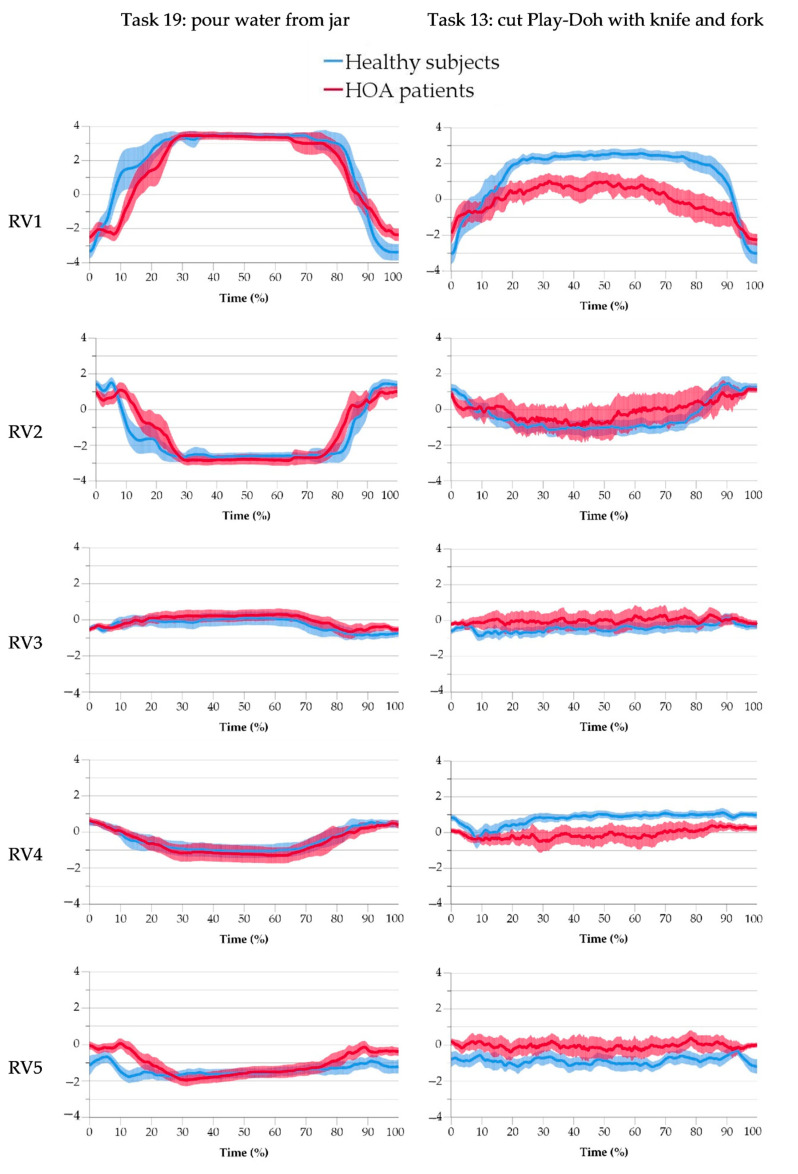
Temporal evolution (mean value across subjects and 95%CI) of RVs in task 19 (pour water from a jar) and task 13 (cut Play Doh with a knife and fork) for healthy subjects (blue) and HOA patients (red).

The temporal evolution of RVs in the two selected representative tasks (task 19: pour water from a jar; and task 13: cut Play-Doh with a knife and fork) is shown in Figure 4. Differences in ranges used by each sample are evident during the knife task in some RVs (RV1, RV4, and RV5), while differences during the gross manipulative task appear only in the times spent on achieving postures, such as in RV1. Note also that task 19 is more repeatable than task 13, which shows high variability across subjects.

#### 3.1.4. Reducing the Number of Sensors

By choosing the joint angle with the highest loading in each synergy of the HOA patients (PPCs in Table 2), the reduced set of joint angles to be measured is as follows: flexion of the metacarpophalangeal joint of the middle finger and the proximal interphalangeal joint of the ring finger, abduction between ring and little fingers, and abduction and flexion of the thumb carpometacarpal joint.

Table 5 shows the RMSDs when estimating the remaining angles with a linear regression, with the thumb IP joint presenting the highest error.

## 4. Discussion

This paper aims to promote the use of kinematics of the entire hand for the assessment of hand function in clinical practice by overcoming current obstacles in the recording and analysis of the high dimensionality problem. We recorded entire hand kinematics of HOA patients and healthy subjects during the performance of ADL by using a commercially available sensorized glove and a calibration method [24] that avoided the need to calibrate the glove for each subject [24], thereby reducing the recording times to a large extent. Additionally, we used dimensional reduction for an in-depth comparison of their kinematics, facilitating the analysis of a high number of degrees of freedom that are used simultaneously by the hand. As a result, this paper presents the most extensive study in HOA patients to date, with the highest number of activities and joints studied, since up until now only a few activities or a few joints in HOA patients have been recorded and analyzed [11,16,17].

Early detection of dysfunction associated to HOA is essential for applying appropriate treatments that can prevent the progression of the disease. To date, only the AROMs in certain affected joints [10,11,12] have been studied as indicators of the degree of compromise. A deeper analysis has been made possible here by combining the use of the Cyberglove and an across-subject calibration protocol [24], which has allowed the recording of 16 DoFs while performing complicated manipulating activities representative of functionality (those from the SHFT) and the analysis of these recorded data by using dimensional reduction.

Dimensionality reduction in hand function assessment has been proven in previous studies [37,38,39] to be a method of detecting the existence of kinematic alterations. By reducing the dimensionality of the kinematics, the results are easier to interpret and could be used in clinical settings. Here, we have proven its feasibility also in HOA patients. We have applied PCA to two samples (healthy and patients), finding five PCs in both cases with a high percentage of variance explained: more than 70%. Synergies obtained for healthy subjects were as follows: *fingers arch* (coordinated flexion of PIP joints of fingers); *hand closure* (coordinated flexion and adduction of MCP joints of fingers); *thumb-index pinch* (coordinated motion of thumb joints and index MCP flexion); *forced thumb opposition* (coordinated abduction of thumb CMC joint with flexion of IP joint and extension of MCP joint of thumb); and *palmar arching* (coordinated palmar arch and thumb CMC abduction). These five synergies are coherent with previous studies: the first two PCs referring to finger MCP and PIP arching [29,42,44,45] and higher order synergies referring to fine manipulation coordination [35]. Patient synergies are similar to the healthy ones for the finger’s arch and hand closure, although the latter explains less variance, which is probably due to MCP compromise in HOA patients [46]. However, patients’ higher order synergies differ from those of healthy subjects. Patients also present a coordination of thumb CMC, MCP, and IP joints but with CMC extension instead of abduction coordinated with the extension of MCP and flexion of IP joints, probably due to thenar atrophy and adduction of the thumb metacarpal joint in HOA patients [47]. They also present a palmar arching coordination, explaining higher variance but more coordinated with the movement of the MCP joint of the little finger than with the thumb, possibly due to opposition difficulties, therefore, resulting in an increased palmar arching instead of forcing the compromised CMC joint [48]. The biggest difference appears in the lack of thumb-index pinch coordination; thus, patients seem to make less use of precision grasps, probably due to compromised index and thumb joints.

Given the similarity of the underlying synergies, we have checked the feasibility of using the healthy ones (HPCs) to reduce the kinematics of both patients and healthy subjects, thus allowing comparison of the two samples in search of possible indicators of kinematics alterations due to HOA pathology. This possibility has been previously applied in feet kinematic analysis during walking by using PCs from normal feet in highly pronated and highly supinated feet [41], with good results. The total amount of variance explained by the HPCs for patients was found to be only slightly smaller than that for healthy subjects (70 (±3) vs. 74 (±2)), thus confirming the feasibility of reducing the kinematics of both samples by using the same HPCs.

In order to look for indicators of kinematic alterations produced by HOA pathology, the kinematics of the two samples were compared in terms of the reduced variables RVi (i = 1 to 5) calculated from the HPCs. Significant differences between samples were found for different summarizing parameters in all RVi except in RV5 (the reduced variable corresponding to *Palmar arching*). Ranges in RV1 to RV4 are significantly different, in accordance with previous studies that found that AROM is reduced in joints affected by HOA [10,11,12] and that the range of motion used in compromised joints is also reduced in certain activities of daily living [11,16,17]. In this study, the ranges used by patients are significantly lower for finger arch coordination, thumb-index pinch, and forced thumb opposition, and they are consistent with limitations in compromised joints (finger PIP joints and thumb joints [47,49]). However, patients present a higher range in hand closure, although with a median hand closure that is less closed (or flexed). Therefore, it seems that these parameters, the ones presenting statistically significant differences, can be used as indicators of HOA pathology. We have proposed a linear discriminant analysis with them that has thrown good results that are much better than a discriminant function based in SHFT scores and times. The discriminant function based on reduced kinematics is able to detect HOA pathology with a success rate of 80%, which is much higher than the detection ability of SHFT. The discriminant function considers the degree of compromise, since moderate and severe cases were correctly classified along with most of the mild ones. This function may also be related to the impact on functionality, since a deficiency in a given kinematic synergy is expected to hinder the performance of tasks that require using this synergy. However, the results from the SHFT set against the difficulties to carry out ADL reported by patients highlights the poor capability of SHFT to discriminate HOA effects on hand function. As a consequence, the relationship with SHFT score cannot be used to measure the degree of disability of HOA patients, and a better scale to measure the impact of HOA on hand function is required.

The comparison of the time evolution of the RVs for the different tasks can be used as specific indicators of the function of the hand in each task, although it needs some considerations. From the two examples included in this paper, some conclusions can be drawn. The first two RVs (fingers arch and hand closure) are highly involved in the ‘pour water from a jar’ task (representative of gross manipulation), because of the use of a cylindrical grasp. High-order synergies requiring thumb-index coordination, palmar arch, and particularly forced thumb opposition are involved in the ‘cutting Play-Doh with a knife and fork’ task, showing the higher manipulative demand of this task. Possible indicators of dysfunction can be inferred by looking for differences in the time profiles. In the gross manipulation task, finger arch (RV1) ranges are similar between samples, although patients achieve the maximum finger arch more slowly. This also happens with hand closure: Patients achieve hand maximum opening more slowly than healthy subjects do. Thus, the time to accommodate finger arch and hand closure synergies in gross manipulative tasks might be used as indicators of dysfunction. Perhaps the reaching velocity in these synergies could be studied as indicators in further studies. In the manipulative task, it is clearly observed that patients use a lower mean fingers arch and a lower forced thumb opposition than compared healthy subjects, but they had higher palmar archings. However, it is difficult to analyze time dependence because of the poor repeatability of the task across subjects, probably due to the different pace needed when cutting the five pieces. A more standardized, shorter task involving cutting with a knife (for example, cutting just one piece) would provide more meaningful information. The analysis of differences in profiles for each RVi, therefore, appears as a promising method of identifying additional kinematic dysfunction indicators that might improve the detection of HOA. However, this search requires the recordings of standardized tasks to be addressed in future studies.

Finally, in order to promote the use of hand kinematics during ADL in clinics, hand synergies in HOA patients have also been used to explore the feasibility of using a very reduced set of joint angles for measurements in order to estimate entire hand kinematics of HOA patients. The errors in estimating the hand joint angles from the measurement of only five joint angles (flexion and abduction of thumb carpometacarpal joint, flexion of metacarpophalangeal joint of the middle finger and proximal interphalangeal joint of the ring finger, and abduction between ring and little fingers) are small (7.0° ± 2.7°). The errors found are of the same order of magnitude as the errors from the recording technique [40]. Despite being only a preliminary exploration, the results obtained suggest the feasibility of acquiring entire hand kinematics from the recording of only a few angles. Further studies are required to make it clear whether the proposed angles are the most suitable ones or if more sophisticated methods for looking for the most appropriate ones [40] would be needed. Searching for indicators of kinematic dysfunction based only on the recordings of these few angles would, therefore, be interesting. This might have a big impact on its usability in clinical settings because of different improvements, such as a reduction in the cost of the device, fewer occultation problems if optical systems were used, and less time required to prepare the patient if sensors must be placed. Even an instrumented glove with only the specific required sensors could be designed.

One limitation of this study is the difference between sample ages and genders. However, it is difficult to say in elderly population if kinematics alterations are due to HOA pathology or only a result of age, since age is main factor of a degenerative pathology, such as arthritis, that may affect subjects that are self-considered as healthy.

## 5. Conclusions

The results obtained provide a function that allows clinicians to detect HOA with a good success rate that is much better than SHFT scores and time scores. Good results pave the way toward obtaining indicators based on healthy synergies underlying SHFT performance for the early detection of HOA that might also detect specific kinematic dysfunctions due to pathology. This has been made possible thanks to the use of an appropriate device, the Cyberglove, with a suitable calibration that allows for recording a high number of DoFs in complicated manipulation tasks and to the application of PCA, which makes it easier to understand and interpret the results. Furthermore, the exploration of how to reduce the number of joints to be recorded has yielded promising results and might become future alternatives for assessing the hand function of HOA patients. Thus, future studies should focus on an in-depth analysis of the indicators proposed during representative standardized and controlled activities. In addition, further analysis in the reduction in required sensors must be performed.

## Figures and Tables

**Table 1:** Activities of daily living considered in the Sollerman hand function test.

Pick coins up from flat surface, put into purses mounted on wall
2. Open/close zip
3. Pick up coins from purses
4. Lift wooden cubes over edge 5 cm in height
5. Lift iron over edge 5 cm in height
6. Turn screw with screwdriver
7. Pick up nuts and turn them until completely screwed onto bolts
8. Put key into Yale lock, turn 90°
9. Turn door-handle 30°
10. Unscrew lid of jars
11. Do up buttons
12. Put Tubigrip stocking on the other hand
13. Cut Play-Doh with a knife and fork
14. Write with pen
15. Fold paper, put into envelope
16. Put paper-clip on envelope
17. Lift telephone receiver, put to ear
18. Pour water from Pure-Pak
19. Pour water from a jar
20. Pour water from a cup

**Table 2:** Loadings of resulting PCs in the PCA performed on each sample. H and P stand for healthy subjects and HOA patients, respectively. For easier interpretation, loadings greater than 0.3 (weak) and 0.5 (strong) are highlighted in light and dark grey, respectively, similarly as in [42]. The last row shows the variance explained by each PC.

		Healthy Subjects					HOA Patients				
Joint	Movement	HPC1	HPC2	HPC3	HPC4	HPC5	PPC1	PPC2	PPC3	PPC4	PPC5
CMC1	Flexion	−0.019	0.109	0.847	0.064	−0.007	−0.016	−0.210	−0.478	−0.115	−0.661
	Abduction	0.387	0.046	0.336	0.499	0.431	0.285	0.099	0.067	0.819	−0.083
MCP1	Flexion	0.089	0.126	0.013	−0.831	0.148	0.188	0.128	0.014	0.082	−0.655
IP1	Flexion	0.114	0.012	−0.571	0.534	0.085	−0.020	0.018	−0.402	0.058	0.476
MCP2	Flexion	0.265	0.728	0.446	−0.064	0.098	0.850	0.165	0.012	0.027	−0.259
MCP3	Flexion	0.333	0.830	0.245	−0.054	0.127	0.916	0.150	−0.072	0.041	−0.102
MCP4	Flexion	0.431	0.823	0.001	0.014	−0.003	0.872	0.202	−0.272	−0.013	0.082
MCP5	Flexion	0.537	0.696	−0.140	0.081	−0.115	0.664	0.226	−0.595	0.089	0.019
PIP2	Flexion	0.633	0.182	−0.028	0.173	0.193	0.183	0.653	0.107	0.050	0.096
PIP3	Flexion	0.934	0.044	−0.045	0.032	0.005	0.106	0.907	−0.014	0.146	0.049
PIP4	Flexion	0.939	0.115	0.011	−0.014	−0.050	0.127	0.934	−0.056	0.073	−0.078
PIP5	Flexion	0.841	0.120	0.038	−0.105	−0.094	0.050	0.873	−0.039	−0.029	−0.103
MCP2−3	Abduction	0.240	−0.622	−0.220	0.112	−0.060	−0.508	0.062	0.012	0.497	0.363
MCP3−4	Abduction	0.191	−0.647	0.199	0.102	0.249	−0.460	0.114	0.308	0.609	0.052
MCP4−5	Abduction	−0.311	−0.521	0.260	−0.129	0.300	−0.171	−0.055	0.817	0.043	0.171
PArch	Flexion	−0.049	−0.064	−0.071	−0.085	0.901	−0.107	0.086	0.693	0.211	−0.132
Variance explained (%)		24.26	22.20	10.00	8.32	7.94	21.60	19.52	13.05	8.74	8.71

**Table 3:** Level of similarity in angles (degrees) between synergies. H and P stand for healthy and pathologic subjects, respectively (lower angles represent more similarity, and angles close to 90° represent no similarity at all).

	HPC1	HPC2	HPC3	HPC4	HPC5
PPC1	64	16	70	87	88
PPC2	19	75	87	90	85
PPC3	74	58	65	77	48
PPC4	69	75	77	69	58
PPC5	87	70	59	41	81

**Table 4:** Statistics of each reduced variable (RVi) summarizing value (median, 5th percentile, 95th percentile, and range as their difference) for both samples, and significant differences between healthy subjects and HOA patients samples are marked with a tick.

			Healthy Subjects				HOA Patients			
RVi		Summarizing Value	Mean	SD	Min	Max	Mean	SD	Min	Max
RV1		Median	0.228	0.212	−0.119	0.636	0.128	0.205	−0.419	0.513
	√	5th percentile	−3.631	0.518	−4.785	−2.627	−3.155	0.394	−4.189	−2.358
		95th percentile	3.147	0.337	2.414	3.861	3.020	0.237	2.549	3.469
	√	Range	6.778	0.430	6.033	7.747	6.176	0.415	5.414	7.189
RV2	√	Median	0.047	0.170	−0.268	0.321	−0.043	0.15	−0.302	0.318
		5th percentile	−2.963	0.248	−3.603	−2.456	−3.047	0.330	−3.723	−2.409
	√	95th percentile	3.282	0.373	2.478	3.924	3.534	0.419	2.757	4.380
	√	Range	6.245	0.456	5.337	7.185	6.582	0.547	5.358	7.679
RV3		Median	−0.121	0.108	−0.350	0.049	−0.087	0.087	−0.236	0.080
	√	5th percentile	−1.915	0.224	−2.408	−1.482	−1.774	0.278	−2.274	−1.253
	√	95th percentile	2.055	0.234	1.686	2.540	1.922	0.27	1.243	2.414
	√	Range	3.971	0.341	3.175	4.541	3.696	0.471	2.698	4.424
RV4	√	Median	0.183	0.096	−0.035	0.326	0.117	0.128	−0.235	0.387
	√	5th percentile	−2.177	0.312	−2.795	−1.635	−1.833	0.387	−2.65	−1.029
	√	95th percentile	1.682	0.211	1.289	2.238	1.550	0.236	1.097	2.094
	√	Range	3.859	0.405	3.128	4.605	3.383	0.496	2.192	4.335
RV5		Median	0.014	0.131	−0.199	0.312	0.027	0.129	−0.181	0.285
		5th percentile	−1.974	0.333	−3.080	−1.512	−1.896	0.269	−2.522	−1.480
		95th percentile	1.797	0.204	1.387	2.249	1.764	0.304	1.244	2.424
		Range	3.771	0.298	3.283	4.538	3.659	0.322	2.880	4.420

**Table 5:** Errors (°) when estimating entire hand kinematics from the recording of joint angle flexions of the metacarpophalangeal joint of the middle finger and the proximal interphalangeal joint of the ring finger, abduction between ring and little fingers, and abduction and flexion of the thumb carpometacarpal joint.

Angle	Flexion									Abduction	
Joint	MCP1	IP1	MCP2	MCP4	MCP5	PIP2	PIP3	PIP5	PArch	MCP2-3	MCP3-4
Error (ᵒ)	6.72	12.39	6.46	6.33	6.78	11.68	6.18	7.08	5.44	4.92	3.35

## References

[1] Van Dokkum L., Hauret I., Mottet D., Froger J., Métrot J., Laffont I. (2014). The contribution of kinematics in the assessment of upper limb motor recovery early after stroke. Neurorehabilit. Neural Repair.

[2] WHO (2001). International Classification of Functioning, Disability and Health (ICF).

[3] Schouffoer A.A., van der Giesen F.J., Beaart-van de Voorde L.J., Wolterbeek R., Huizinga T.W., Vliet Vlieland T.P. (2016). Validity and responsiveness of the michigan hand questionnaire in patients with systemic sclerosis. Rheumatology.

[4] Dias J.J., Rajan R.A., Thompson J.R. (2008). Which questionnaire is best? The reliability, validity and ease of use of the patient evaluation measure, the disabilities of the arm, shoulder and hand and the michigan hand outcome measure. J. Hand Surg. Eur. Vol..

[5] Dalton E., Lannin N.A., Laver K., Ross L., Ashford S., McCluskey A., Cusick A. (2017). Validity, reliability and ease of use of the disabilities of arm, shoulder and hand questionnaire in adults following stroke. Disabil. Rehabil..

[6] Sears E.D., Chung K.C. (2010). Validity and responsiveness of the jebsen-taylor hand function test. J. Hand Surg. Am..

[7] Sollerman C., Ejeskär A. (1995). Sollerman hand function test: A standardised method and its use in tetraplegic patients. Scand. J. Plast. Reconstr. Surg. Hand Surg..

[8] Light C.M., Chappell P.H., Kyberd P.J. (2002). Establishing a standardized clinical assessment tool of pathologic and prosthetic hand function: Normative data, reliability, and validity. Arch. Phys. Med. Rehabil..

[9] Reyes-Guzmán A.D.L., Dimbwadyo-Terrer I., Trincado-Alonso F., Monasterio-Huelin F., Torricelli D., Gil-Agudo A. (2014). Quantitative assessment based on kinematic measures of functional impairments during upper extremity movements: A review. Clin. Biomech..

[10] Villafañe J.H., Valdes K. (2013). Combined thumb abduction and index finger extension strength: A comparison of older adults with and without thumb carpometacarpal osteoarthritis. J. Manip. Physiol. Ther..

[11] Holland S., Straatman L., MacDermid J., Sinden K., Lalone E. (2020). The development of a novel grip motion analysis technique using the dartfish movement analysis software to evaluate hand movements during activities of daily living. Med. Eng. Phys..

[12] Kroon F.P.B., Damman W., Liu R., Bijsterbosch J., Meulenbelt I., van Der Heijde D., Kloppenburg M. (2018). Validity, reliability, responsiveness and feasibility of four hand mobility measures in hand osteoarthritis. Rheumatology.

[13] Dahaghin S., Bierma-Zeinstra S.M., Ginai A.Z., Pols H.A.P., Hazes J.M.W., Koes B.W. (2005). Prevalence and pattern of radiographic hand osteoarthritis and association with pain and disability (the rotterdam study). Ann. Rheum. Dis..

[14] Haugen I.K., Englund M., Aliabadi P., Niu J., Clancy M., Kvien T.K., Felson D. (2011). Prevalence, incidence and progression of hand osteoarthritis in the general population: The framingham osteoarthritis study. Ann. Rheum. Dis..

[15] Hermann W., Lambova S., Muller-Ladner U. (2018). Current treatment options for osteoarthritis. Curr. Rheumatol. Rev..

[16] Luker K.R., Aguinaldo A., Kenney D., Cahill-Rowley K., Ladd A. (2014). Functional task kinematics of the thumb carpometacarpal joint. Clin. Orthop. Relat. Res..

[17] Tanashi A., Szekeres M., MacDermid J., Lalone E.A. (2021). Comparison of finger kinematics between patients with hand osteoarthritis and healthy participants with and without joint protection programs. J. Hand Ther..

[18] Fu Q., Santello M. (2011). Towards a complete description of grasping kinematics: A framework for quantifying human grasping and manipulation. Proceedings of the 2011 Annual International Conference of the IEEE Engineering in Medicine and Biology Society.

[19] Cescon C., Tettamanti A., Barbero M., Gatti R. (2015). Finite helical axis for the analysis of joint kinematics: Comparison of an electromagnetic and an optical motion capture system. Arch. Physiother..

[20] Buffi J.H., Crisco J.J., Murray W.M. (2013). A method for defining carpometacarpal joint kinematics from three-dimensional rotations of the metacarpal bones captured in vivo using computed tomography. J. Biomech..

[21] Eccarius P., Bour R., Scheidt R. (2012). Dataglove measurement of joint angles in sign language handshapes. Sign Lang. Linguist..

[22] Griffin W.B., Findley R.P., Turner M.L., Cutkosky M.R. Calibration and mapping of a human hand for dexterous telemanipulation. Proceedings of the Asme Imece 2000 Symposium on Haptic Interfaces for Virtual Environments and Teleoperator Systems.

[23] Kessler G.D., Hodges L.F., Walker N. (1995). Evaluation of the cyberglove as a whole-hand input device. ACM Trans. Comput.-Hum. Interact..

[24] Gracia-Ibáñez V., Vergara M., Buffi J.H., Murray W.M., Sancho-Bru J.L. (2017). Across-subject calibration of an instrumented glove to measure hand movement for clinical purposes. Comput. Methods Biomech. Biomed. Eng..

[25] Roda-Sales A., Vergara M., Sancho-Bru J.L., Gracia-Ibáñez V., Jarque-Bou N.J. (2019). Effect on hand kinematics when using assistive devices during activities of daily living. PeerJ.

[26] Jarque-Bou N.J., Vergara M., Sancho-Bru J.L., Gracia-Ibanez V., Roda-Sales A. (2020). Hand kinematics characterization while performing activities of daily living through kinematics reduction. IEEE Trans. Neural Syst. Rehabil. Eng..

[27] Daffertshofer A., Lamoth C.J.C., Meijer O.G., Beek P.J. (2004). Pca in studying coordination and variability: A tutorial. Clin. Biomech. Bristol. Avon..

[28] Witte K., Ganter N., Baumgart C., Peham C. (2010). Applying a principal component analysis to movement coordination in sport. Math. Comput. Model. Dyn. Syst..

[29] Santello M., Flanders M., Soechting J.F. (1998). Postural hand synergies for tool use. J. Neurosci..

[30] Mason C.R., Gomez J.E., Ebner T.J. (2001). Hand synergies during reach-to-grasp. J. Neurophysiol..

[31] Della Santina C., Bianchi M., Averta G., Ciotti S., Arapi V., Fani S., Battaglia E., Catalano M.G., Santello M., Bicchi A. (2017). Postural hand synergies during environmental constraint exploitation. Front. Neurorobot..

[32] Thakur P.H., Bastian A.J., Hsiao S.S. (2008). Multidigit movement synergies of the human hand in an unconstrained haptic exploration task. J. Neurosci..

[33] Portnova-Fahreeva A.A., Rizzoglio F., Nisky I., Casadio M., Mussa-Ivaldi F.A., Rombokas E. (2020). Linear and non-linear dimensionality-reduction techniques on full hand kinematics. Front. Bioeng. Biotechnol..

[34] Cui P.H., Visell Y. (2014). Linear and nonlinear subspace analysis of hand movements during grasping. Proceedings of the 2014 36th Annual International Conference of the IEEE Engineering in Medicine and Biology Society.

[35] Gracia-Ibáñez V., Sancho-Bru J.L., Vergara M., Jarque-Bou N.J., Roda-Sales A. (2020). Sharing of hand kinematic synergies across subjects in daily living activities. Sci. Rep..

[36] Gracia-Ibáñez V., Vergara M., Sancho-Bru J.L. Human hand synergies in activities of daily living. Proceedings of the 22th Congress of the European Society of Biomechanics.

[37] Gracia-Ibáñez V., Sancho-Bru J., Vergara M., Mottet D., Laffont I., Bahkti K. Feasibility of hand function assessment through kinematics reduction in different pathologies. Proceedings of the 23rd Congress of the European Society of Biomechanichs.

[38] Gracia-Ibáñez V., Sancho-Bru J., Vergara M., Mottet D., Laffont I., Bahkti K., Llop-Harillo I., Pérez-González A. Mimicking kinematics synergies underlying activities of daily living for rehabilitation. Proceedings of the 11th Symposium of Hand and Wrist Biomechanics International in Conjunction with the International Society of Biomechanics Congress XXVI.

[39] Gracia-Ibáñez V., Sancho-Bru J., Vergara M., Mottet D., Laffont I., Bahkti K. Hand function assessment of patients with stroke through reduced kinematic analysis. Proceedings of the Engineering the Upper Limb Institution of Mechanical Engineers.

[40] Jarque-Bou N., Sancho-Bru J., Vergara M. (2021). Synergy-based sensor reduction for recording the whole hand kinematics. Sensors.

[41] Sanchis-Sales E., Rodríguez-Cervantes P., Sancho-Bru J. (2019). Kinematics reduction applied to the comparison of highly-pronated, normal and highly-supinated feet during walking. Gait Posture.

[42] Jarque-Bou N., Gracia-Ibáñez V., Sancho-Bru J., Vergara M., Pérez-González A., Andrés F. (2016). Using kinematic reduction for studying grasping postures. An application to power and precision grasp of cylinders. Appl. Ergon..

[43] Singh H.P., Dias J.J., Thompson J.R. (2015). Timed sollerman hand function test for analysis of hand function in normal volunteers. J. Hand Surg. Eur. Vol..

[44] Jarque-Bou N.J., Scano A., Atxori M., Müller H. (2019). Kinematic synergies of hand grasps: A comprehensive study on a large publicly available dataset. J. Neuroeng. Rehabil..

[45] Ingram J.N., Körding K.P., Howard I.S., Wolpert D.M., Kording K.P., Howard I.S., Wolpert D.M. (2008). The statistics of natural hand movements. Exp. Brain Res..

[46] Kouki I., Tuffet S., Crema M.D., Rousseau A., Richette P., Dougados M., Berenbaum F., Sellam J., Courties A. (2021). Metacarpophalangeal impairment in hand osteoarthritis is not rare and is associated with mechanical factors: Results from the digicod hand osteoarthritis cohort. Arthritis Care Res. Hoboken.

[47] Kloppenburg M., van Beest S., Kroon F.P.B. (2017). Thumb base osteoarthritis: A hand osteoarthritis subset requiring a distinct approach. Best Pract. Res. Clin. Rheumatol..

[48] Gehrmann S.V., Tang J., Li Z.M., Goitz R.J., Windolf J., Kaufmann R.A. (2010). Motion deficit of the thumb in cmc joint arthritis. J. Hand Surg. Am..

[49] Camacho Encina M., Calamia V., Picchi F., Ortega I., Fernández Puente P., González Rodríguez L., Ruiz Romero C., Blanco F.J. (2018). Fri0520 discovery of potential biomarkers for the diagnosis of erosive and nodal hand osteoarthritis. Ann. Rheum. Dis..

